# Density Matching for Microencapsulation of Field Responsive Suspensions of Non-Brownian Microparticles

**DOI:** 10.1021/acs.jpcb.4c02288

**Published:** 2024-05-23

**Authors:** Samuel R. Wilson-Whitford, Jinghui Gao, James F. Gilchrist

**Affiliations:** † Department of Chemical and Biomolecular Engineering, Lehigh University, Bethlehem, Pennsylvania 18015, United States; ‡ School of Engineering, The University of Warwick, Coventry CV4 7AL, U.K.

## Abstract

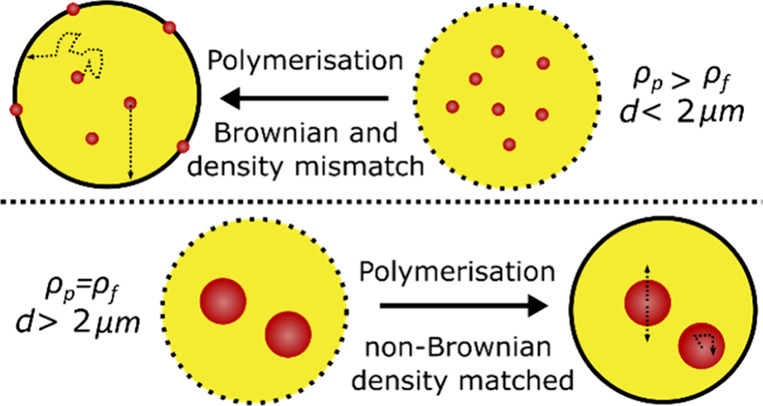

When forming composite microcapsules through the emulsification of a dispersed phase laden with microparticles, one will find that the microparticles become irreversibly embedded in the resulting microcapsule membrane. This phenomenon, known as Pickering stabilization, is detrimental when the end function of the microcapsules relies on the mobility of encapsulated microparticles within the capsule core. In this work, a robust microencapsulation route using density matching of non-Brownian microparticles in a binary solvent is shown to easily and effectively encapsulate particles, with >90% of particles retaining mobility within the microcapsules, without the necessity for prior chemical/physical modifications to the microparticles. This is proposed as a generalized method to be used for all manner of particle chemistries, shapes, and sizes.

## Introduction

Microcapsules containing microparticles, either at the interface or contained within the core of capsules, are a type of composite material capable of combining the desirable properties of their component materials. Encapsulated particles can have any number of chemistries, with the microcapsule itself providing either a structural template or compartmentalization vessel for what are usually expensive microparticle additives. Combinations of capsules and particles can be used to control capsule structural properties^1^ and release dynamics,^2−4^ or used in analytics,^5^ as microreactors,^6^ responsive systems,^7^ and electrochemical and absorption micromaterials,^8−10^ in a wide variety of pharmaceutical, environmental, and energy applications.

Typically, emulsification in the presence of microscale particles can result in adsorption of particles to the droplet interface, in a process called Pickering stabilization.^11−13^ There is a significant energetic advantage to solid particles occupying the interface.^14,15^ The presence of particles in the dispersed phase during microencapsulation has a tendency to exacerbate this phenomenon, with particles almost instantaneously stabilizing the interface, even under laminar conditions in microfluidic experiments.^16^ Although there are a wealth of applications for particle-stabilized/armored capsules, there are of course examples where the desired result is for particles to be freely suspended or mobile inside the core of microcapsules.

One of the most convenient and intuitive approaches to minimize particle adsorption to the interface is to significantly restrict the Brownian motion of the encapsulant particles during the microencapsulation process. This is done typically through control of the rheology of the core.^17−20^ However, in most cases, the gelation of the core will be irreversible and will permanently restrict the mobility of encapsulant particles, which in the context of this discussion is counterproductive. Likewise, yield stress and viscoelastic fluids increase complexity with regard to scale-up. Lim and Moss demonstrated a series of techniques for encapsulating biologicals using an aqueous reliquification approach making use of ionic cross-linking and chelation agents.^21−23^ Previously, the use of a yield stress core material that was sufficiently weak as to yield under externally imparted forces, facilitating particle motion, was demonstrated for suspensions of encapsulated particles.^24^

Generation of particles *in situ* within the core of capsules has been shown using variations on emulsion polymerization^25−29^ and template-free approaches,^30,31^ but these approaches are often limited to producing capsules where the core particles and capsule wall are made of the same material. Alternatively, layer-by-layer approaches in which the encapsulant particle is first coated in a sacrificial layer, which can later be removed following shell formation,^32−37^ are capable of producing highly monodisperse materials, at the cost of being synthetically laborious. Additionally, there are approaches that rely on utilizing complementary surface energies or chemical modification of particles.^38−40^ In circumstances where the experimental and/or product parameters are strict, the use of surface modification of particles is an effective methodology for encapsulating mobile particles. However, each new formulation will require a similar degree of experimentation and optimization.

**Scheme 1:**
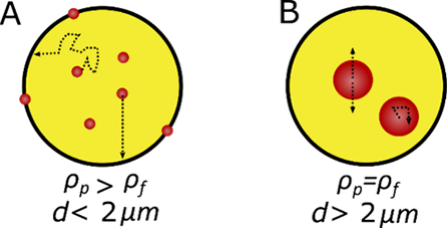
Schematic of (A) Particle Adsorption to Microcapsule Membranes in a Typical Microencapsulation of Suspended Particles. (B) Proposed Microencapsulation of Density Matched Non-Brownian Microparticles In the scheme, *d* refers to the particle diameter, ρ_p_ is the particle density, and ρ_f_ is the fluid density.

Additionally, it is not always practical or possible to modify the surface properties and wetting of the particles that will be encapsulated or to conduct extensive solvent searches for surface energy matching. Likewise, the solidification of cores or the modification of the core viscosity can also be restrictive to applications. Therefore, there is a need for a direct, easily scalable approach. This can be especially relevant for industrial applications where the synthesis of custom particles can be prohibitively expensive. Here, it is proposed that provided a particle is neutrally buoyant, preventing sedimentation, and sufficiently large so that Brownian diffusion is insignificant, the time for particle migration to the interface will be greater than the time of membrane polymerization, therefore maintaining a high proportion of mobile particles in the compartmentalized suspensions (Scheme 1). This method is a generalized synthetic approach that is applicable to a range of particle sizes, generally >2 μm diameter, shapes, and chemistries of variable wetting and having moderate density.

## Methods

### Janus Particle Synthesis

The synthetic method for preparation of the 50 μm Janus particles used in this article is previously outlined in full in another publication.^41^ In short, monolayers of ∼45 μm poly(methyl methacrylate) particles were prepared by using an automated Langmuir–Blodgett (ALB) method.^42^ These monolayers were coated with iron, which in turn oxidized to iron oxide, via treatment with physical vapor deposition (PVD). The resulting Janus particles were removed from the substrate in ethanol using sonication and subsequently dried.

### Microcapsule Synthesis

The full list of experimental compositions is given in Table 1. An example synthesis for sample 3 is given here: In a 250 mL beaker, a 2 wt % solution of Mowiol 8-88 PVA was prepared and heated to 45 °C. An impeller stirrer was positioned centrally in the beaker, and the solution was stirred at 450 rpm. In a 20 mL glass vial, 7.5 mL of the binary solvent was prepared by using 3.00 mL of dodecane and 6.00 g of halocarbon 0.8 oil. To this binary solvent 0.018 g of Span 80, 0.089 g of Janus particles, and 625 μL of isophorone diisocyanate were added. The density of the dispersed phase was adjusted dropwise with halocarbon or dodecane until Janus particles were neutrally buoyant. The suspension was sonicated for 5 min and was then quickly added into the stirring continuous phase and left to stir at 500 rpm for 20 s. Immediately following this, the stir speed was adjusted to 150 rpm, and 3 mL of 10 w/w diethylene triamine solution was added dropwise over 30 s. The reaction was left for 4 h, resulting in a microcapsule suspension. For analysis, monolayers of dried capsules were prepared and agitated using a magnetic stir plate, and data was collected via microscopy with a confocal microscope and a USB microscope.

**Table 1:** Composition of Components in the Microcapsule Samples

sample	*V*_Binary_ [cm^3^]	ϕ_p,disp_	*m*_particles_ [g]	*m*_span_ [g]
0	7.5	0	0	0
1	7.5	2.5 × 10^–3^	0.022	0.004
2	7.5	5.0 × 10^–3^	0.044	0.009
3	7.5	7.5 × 10^–3^	0.089	0.018
4	7.5	1.0 × 10^–2^	0.273	0.055
5	7.5	3.0 × 10^–2^	0.465	0.093
6	7.5	5.0 × 10^–2^	0.717	0.144

### Data Analysis

Data was collected by videography for static and agitated microcapsule samples. Data analysis was performed using established image analysis techniques. Details of equipment are listed in the Supporting Information file.

## Results and Discussion

Density matching by the use of binary solvents is convenient as it gives an incredibly broad range of microparticle types that can be encapsulated. Density matching in combination with specific particle modifications has been shown in E-ink synthesis for Brownian scale microparticles.^43−45^

If a particle adheres to the interface before membrane polymerization is sufficiently progressed, then it will become permanently embedded in the capsule wall. Therefore, the time taken for particles to reach the interface, *t*, must be more than the time taken for membrane formation. The time scale of mobility of Brownian or near-Brownian particles toward the interface can be described with the relationship *t* ∼ *L*^2^/*D*, where *L* is the distance of a particle from the interface and *D* is the particle diffusivity. Here, *D* ∝ *r*^–1^, where *r* is the particle radius (eq 1) and therefore the time taken to move to the interface, *t* ∝ *r*.
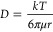
1

However, as the particle size increases and buoyancy and sedimentation become more significant, the time for particles to reach the interface is inversely proportional to the settling velocity, *v*. As governed in eq 2, increasing the particle radius for a fixed particle density, ρ_p_, and fluid viscosity, μ, will decrease *t* such that *t* ∝ *r*^–2^.
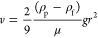
2

As a result, the use of density matching, such that the density difference between the particles and the suspending fluid tends to zero, (ρ_p_ – ρ_f_) → 0, minimizes the buoyant and gravitational components of the particle motion, slowing particle transport toward the polymerizing interface, *t* ∝ 1/(ρ_p_ – ρ_f_). Hence, the development of a typical polymeric encapsulation system utilizing both larger particles and density matching would be a synthetic approach with an inherent resistance to particle adsorption.

Providing that solvents are fully miscible, the density can be adjusted for any value between the density of solvent 1, ρ_1_, and solvent 2, ρ_2_. This is especially convenient when considering blends of materials such as low-density mineral oils and higher density halogenated oils, specifically those that are fluorinated. Here, a binary solvent system consisting of dodecane (ρ_1_ = 0.75 g cm^–3^) and halocarbon 0.8 oil (ρ_2_ = 1.71 g cm^–3^) was used. The halocarbon oils are a range of low molecular weight, oligomeric fluorinated oils based around polychlorotrifluoroethylene (PCTFE), with halocarbon 0.8 oil chosen due to its high density and low room temperature viscosity (0.8 cSt). Figure S1, Supporting Information shows the density curve for blends of dodecane and halocarbon oil, calculated volumetrically.

Poly(methyl methacrylate) (PMMA) particles with an approximate diameter of 35–55 μm (Figures S2 and S3)^41^ were chosen due to their density (∼1.18 g cm^–3^), which lies within a density range (0.75–1.71 g cm^–3^) that is easily adjusted for with conventional solvents. Provided that the density of a particle also lies within the same range, any large microparticle could be used. Referring back to the density curve, a solvent ratio of ∼2:1 g:g of halocarbon:dodecane can be used to approximate density matched PMMA microparticles.

Magnetic Janus particles are used within this example of the synthesis as the particles can easily be manipulated by an external field to demonstrate that they are not adsorbed to the capsules’ walls. The dual hemisphere nature of the particles highlights the translational and rotational freedom of the microparticles inside the microcapsules. A secondary advantage of using Janus particles is that they also demonstrate the robustness of this method toward microparticles with different surface chemistries. The specific particles here are half-coated with a thin 100 nm iron coating. This thin layer would only have a slight influence of the particle density (<0.02 g cm^–3^) and was not included in the approximate particle density. A small amount of Span 80 was also used to reduce particle aggregation.^46^

**Scheme 2:**
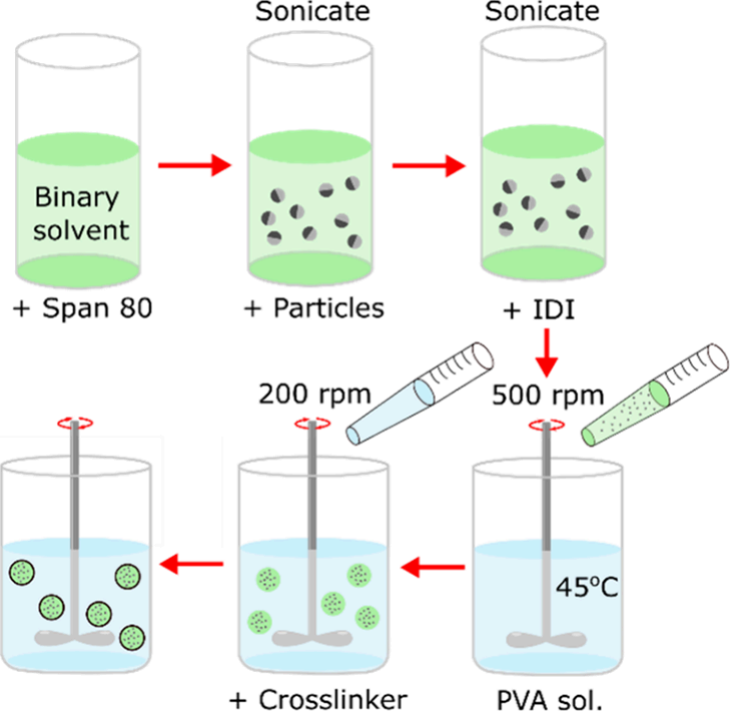
Scheme of the Reaction Process for Microencapsulation of Neutrally Buoyant Particles Green liquid indicates dispersed phase preparation, and blue represents the aqueous continuous phase.

The synthetic method is shown in Scheme 2 and discussed in the Methods section and Table 1. The procedure follows a simple emulsification of the dispersed phase containing the responsive particles. The reaction proceeds via an interfacial polymerization between oil-soluble isophorone diisocyanate (IDI) in the dispersed phase and water-soluble diethylene triamine (DETA) cross-linker added to the continuous phase, producing a polyurea capsule. The dispersed phase contains a volume fraction of Janus particles, ϕ_p, disp_, between 2.5 × 10^–3^ and 5.0 × 10^–2^. The size distributions of the capsules are shown in Figure S4. The mean diameter of the capsules reduces, and the distribution narrows with increased ϕ_p, disp_ as the large particles increase the shear rate during emulsification. Capsule diameters range between ∼450 and 265 μm. The broad distributions are a result of the emulsification method. Additionally, during the synthesis, if the stir rate remains high after the addition of a cross-linker, then the capsules will be destroyed in the early stages of the reaction.

**Figure 1:**
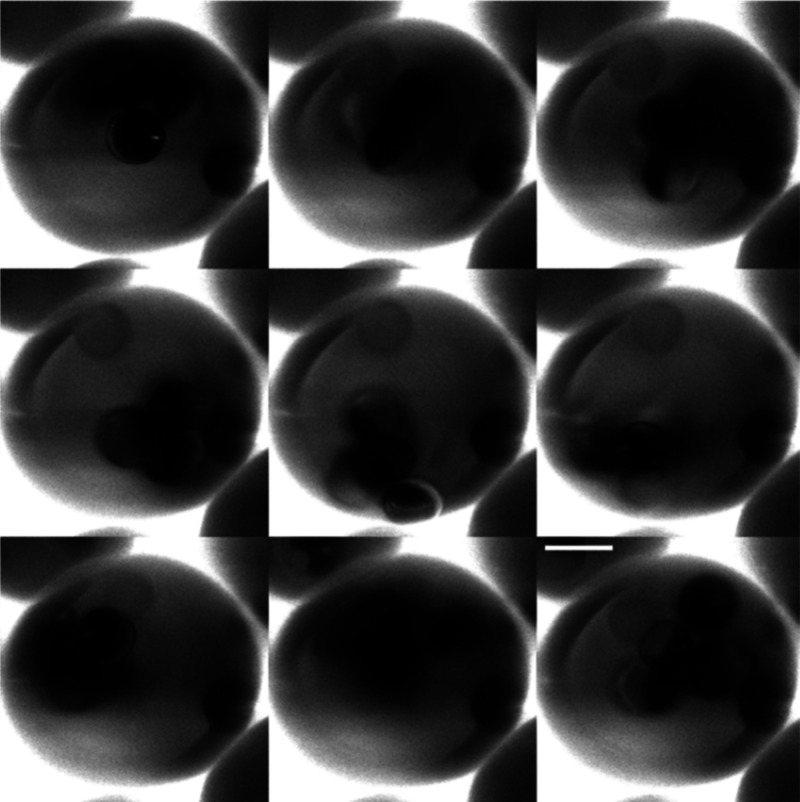
Montage of confocal microscopy images of a single microcapsule containing Janus particle suspension under agitation by an external field. Total duration = 4 s; images at 0.5 s intervals. Scale bar = 50 μm. ϕ_p, disp_= 5.0 × 10^–2^.

The synthesized capsules were exposed to an external actuating magnetic field (∼1 to 8 mT) to assess the population of spatially manipulatable particles within each capsule. Figure 1 and Movie S1 show a time-evolved image of a microcapsule containing Janus particles for a bulk particle concentration ϕ_p, disp_= 5 × 10^–2^. Over a 4 s period, particles are shown to freely rotate and translate throughout the core of the capsule. It should be noted that a particle embedded in the capsule wall will not be released by using a magnetic field. A lower magnification movie of a sample of ϕ_p, disp_= 3.0 × 10^–2^ is also shown in Movie S2.

**Figure 2:**
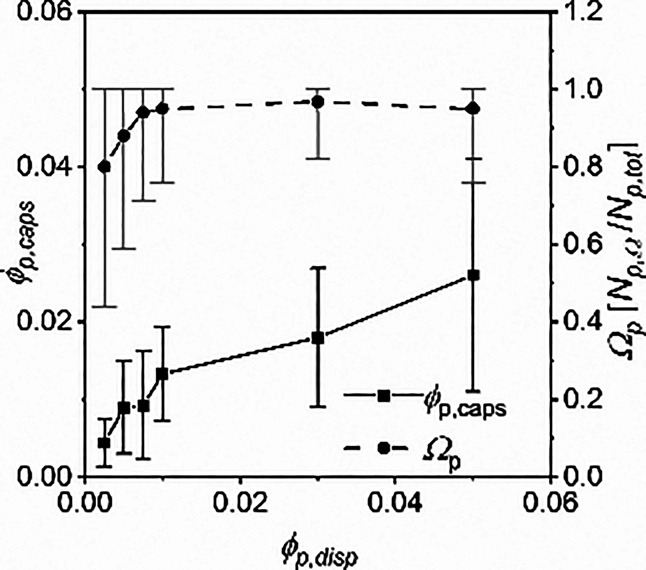
(Black line, black squares) Volume fraction of magnetic particles inside capsules, ϕ_p, caps_, against the initial dispersed phase volume fraction, ϕ_p, disp_. (Dash line, black circles) Fraction of particles within a capsule, which move under applied field manipulation, Ω_p_. Measurements were taken per capsule for a minimum of 300 capsules.

The number of particles per capsule, *N*_p_, volume fraction of particles per capsule, ϕ_p, caps_, and fraction of mobile particles per capsule, Ω_p_, are shown in Table S1 of the Supporting Information, and results for Ω_p_ and ϕ_p, caps_ are represented in Figure 2. Note that Ω_p_ = *N*_Ω, p_/*N*_p, total_ where *N*_Ω, p_ is the number of moving particles per capsule and *N*_p, total_ is the total number of particles per capsule including those embedded in the membrane. As expected, *N*_p_ increases with increased ϕ_p, disp_ but begins to decrease at ϕ_p, caps_= 5.0 × 10^–2^ as the reducing average capsule size decreases the available space within capsules to hold particles. For ϕ_p, caps_vs ϕ_p, disp_, initially, the slope of the trend is >1, suggesting that the tendency of particles to dimerize or chain locally increases the particle concentration for some droplets during emulsification. From ϕ_p, disp_= 1.0 × 10^–2^ onward, the slope is <1, suggesting that a fraction of particles are being lost to the aqueous phase during emulsification, indicating a decreased efficiency in the encapsulation process, possibly due to droplet overcrowding or enlarged cluster sizes. As ϕ_p, disp_ increases, the fraction of mobile particles per capsule, Ω_p_, increases to close to 1 as the statistical significance of capsules containing single immobile particles is nullified. For this system, trends suggest an optimal synthesis at ϕ_p, disp_= ∼2.0 × 10^–2^ where the ϕ_p, disp_≈ ϕ_p, caps_, which also coincides well with close to Ω_p, max_. From a global perspective, for a different particle-capsule system, the results suggest an optimization based on capsule overcrowding and optimal ϕ_p, caps_.

This synthetic technique has also been effective for smaller particle sizes, such as 3, 5, and 8 μm polystyrene particles and also 9–13 μm glass particles and nickel flakes. However, there is visibly reduced encapsulation efficacy and Ω_p_ for smaller particle sizes (*d* ≤ 2 μm) where Brownian motion plays a more significant role.

**Figure 3:**
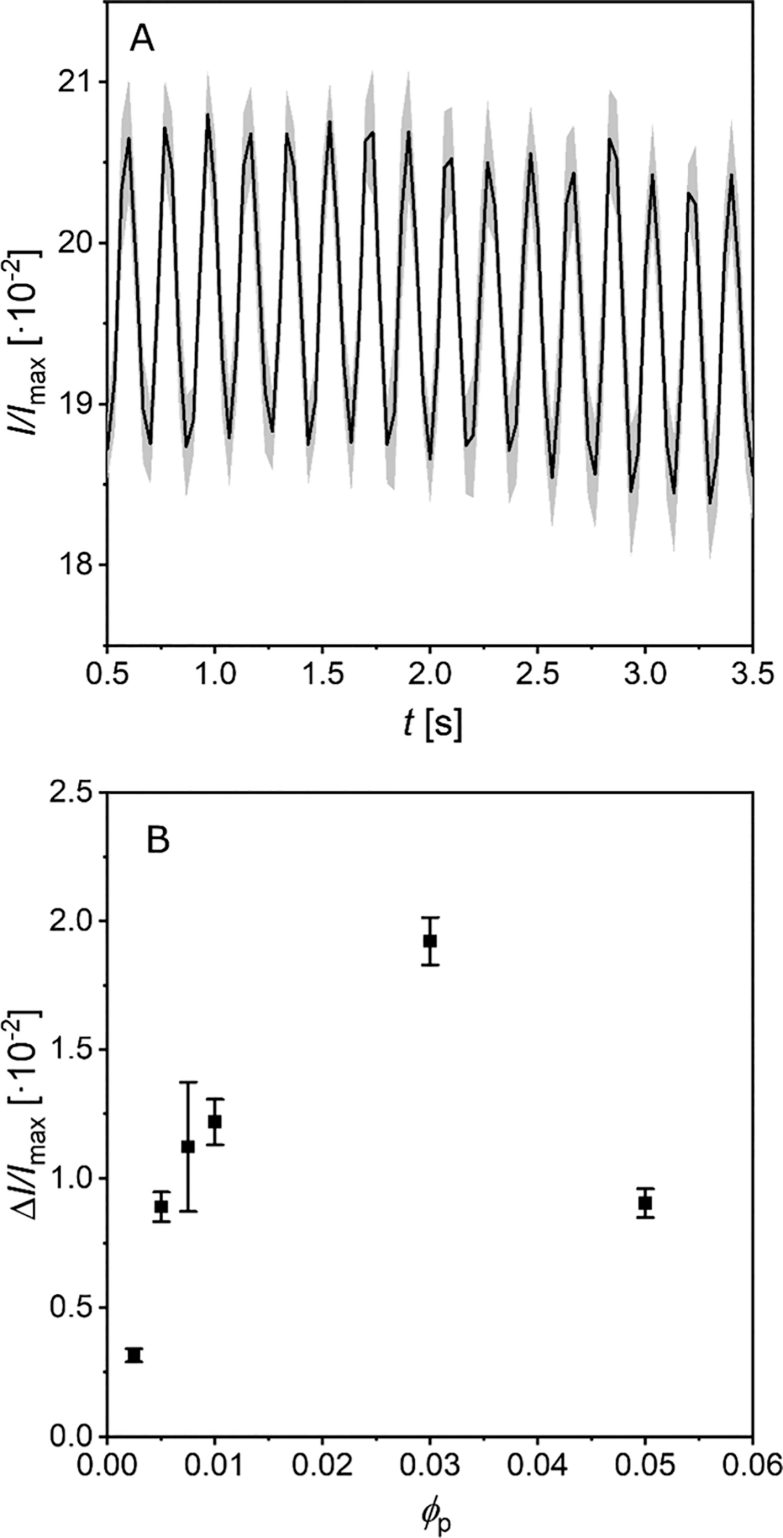
(A) Relative intensity difference of capsules in a rotating magnetic field for a ϕ_p, disp_= 3.0 × 10^–2^. (B) Δ*I*/*I*_max_ for capsules prepared from dispersed phases with increasing ϕ_p, disp_.

To demonstrate the utility of this synthetic method to general applications, we can utilize one of the inherent properties of Janus particle suspensions, as shown previously by Gao et al.^47^ Janus particle suspensions show an optical dynamic range, depending on their orientation and degree of chaining. Among the many possible applications discussed in the Introduction, one which is particularly applicable to this observation is magnetically actuated display materials. Other work also discusses the optical dynamic range of suspensions of suspended magnetic particles.^48^ Specifically, for the work of Gao and co-workers, the orientation of assembled Janus particle chains and clusters relative to an applied magnetic field gives rise to variable transmission of light, essentially as a consequence of the projected surface area of assembled particles. The use of larger Janus particles was also shown to be useful in screen-like devices, though these approaches did not involve microencapsulation.^49−51^ Compartmentalization of these particle suspensions produces microcapsules that display the same optical dynamic properties. The advantages here would be the lack of requirement for a transparent electrode, thus reducing device expense and fragility, yet also giving an order of magnitude increase in the refresh rate of rotating magnetic particles compared to translational motion of particles, such as those seen in established E-inks.^43^Figure 3 shows the results of the analysis of relative intensity difference of these microcapsules at different ϕ_p, disp_ as they are subjected to a variable magnetic field, but an example of the macroscopic phenomenon can be seen in Movie S3. Figure 3A shows the relative intensity difference for capsules prepared from ϕ_p, disp_= 3.0 × 10^–2^ for a magnetic field changing at a frequency of ∼4.8 Hz. Figure 3B shows the difference between *I*_min_ and *I*_max_ over every magnetic pole switch, averaged over 4 s for each ϕ_p, disp_. The measured intensity difference is in the range of 0.31–1.92% and is comparable to that shown in a previous study with smaller particles.^24^ This range depends on several material parameters that remain far from being optimized in this study. The trend in Δ*I*/*I*_max_ increases with ϕ_p, disp_ up to 3.0 × 10^–2^, which is comparable to the synthetic optimization discussed earlier. Δ*I*/*I*_max_ then decreases. This can be explained in two ways. First, as discussed, the orientation of Janus particles is the phenomenon that produces the intensity difference through the changing projected surface area of particles, particle chains, and particle clusters, which in turn changes the transmission/reflection of light through a capsule. As ϕ_p, caps_ increases, the particles are covering an increasing projected surface area. There will be a critical ϕ_p, caps_ at which Δ*I*/*I*_max_ is optimized. At concentrations higher than this, overcrowding inside capsules and particles lost into the aqueous phase remains in the dried samples preventing passage of light through the samples, and Δ*I*/*I*_max_ reduces. Second, overcrowding in capsules can disrupt capsule membrane formation. This results in capsule surface defects, which affect the refractive index difference of the capsule membranes, leading to a slight loss of transparency in the visible range. These are two mechanisms that can be optimized by altering particle size, reflectivity and absorptance, volume fraction, and the background and lighting used.

## Conclusions

In conclusion, density matching of non-Brownian microparticles with a binary dispersed phase has been shown to be an effective route to microencapsulation of microparticles, importantly with a high percentage of particles remaining mobile within the capsule cores when exposed to an external stimulus. The method is also seen to be resistant to particles that have complex/asymmetric wetting properties. The general utility of the method is demonstrated by incorporating magnetoresponsive Janus particles inside the cores of the capsules. The aim is to retain the previously reported optical properties of Janus particle suspensions after microencapsulation. When exposed to a variable magnetic field, the relative transmission of light through the sample oscillates in accordance with findings from the literature but is importantly a visually tangible demonstration of the retention of particle mobility within the capsules without modification of the suspending fluid rheology. The optimal synthetic procedure for maximizing microparticle mobility is comparable to the optimized procedure found for preserving optical activity, showing the robustness of this general procedure.

## Supporting Information Available

Information regarding equipment and materials, particle characterization, and additional capsule characterization (PDF)
Movie 1: Counterpart movie to Figure 1, confocal microscopy recording of particles being agitated inside the core of a capsule (AVI)
Movie 2: Low magnification recording of capsules containing particles under magnetic agitation (AVI)
Movie 3: Macroscopic recording of slurry of wet capsules demonstrating variable optical properties under magnetic agitation (AVI)

## Supplementary Material

jp4c02288_si_001.pdf

jp4c02288_si_002.avi

jp4c02288_si_003.avi

jp4c02288_si_004.avi
