# Supplementary material for: Density Matching for Microencapsulation of Field Responsive Suspensions of Non-Brownian Microparticles
Source: J Phys Chem B. 2024 May 23;128(26):6394–9. doi: 10.1021/acs.jpcb.4c02288 (PMC11228997; doi:10.1021/acs.jpcb.4c02288)
Supplement: Supplementary file 1 — jp4c02288_si_001.pdf [file jp4c02288_si_001.pdf]

Supporting Information: Density Matching for Microencapsulation of Field Responsive Suspensions of non-Brownian Microparticles

Samuel R. Wilson-Whitford[a,b], Jinghui Gao[a] and James F. Gilchrist*[a]*

[a]Department of Chemical and Biomolecular Engineering, Lehigh University Bethlehem, Pennsylvania, USA, 18015

[b]School of Engineering, The University of Warwick, Coventry, UK, CV4 7AL

Email Correspondence: samuel.wilson-whitford@warwick.ac.uk

Email Correspondence: gilchrist@lehigh.edu

Materials

Dodecane (>99%), Mowiol 8-88 (Mw = 67 g mol-1) polyvinyl alcohol and Rhodamine 6G were purchased from Sigma-Aldrich. Isophorone diisocyanate (98%) was purchased from MP Biomedicals and diethylene triamine (99%) was purchased from Alfa Aesar. Span 80 was purchased from Merck. Halocarbon 0.8 oil was provided free of charge from Halocarbon, USA. 38-53 µm PMMA particles were purchased from Syringia lab supplies.

Equipment

Automatic Langmuir-Blodgett deposition set up detailed in previous publication as reference in the main text. Suspensions were sonicated using a Fisher Scientific FS20D. Volume measurements were performed with calibrated 100 μL and 1000 μL Fisherbrand Elite pipettes. Vapor deposition was performed using Eddy SC 20 E-Beam Evaporator. Microscopy of samples was performed on a Visitech International confocal microscope fitted with a VT-eye high speed AOD based point scanner and 20x objective. Emulsification was performed with an IKA impeller stirrer fitted with a 3 blade stirrer, ~45 mm diameter.

Movie 1. Recording of single capsule containing responsive particles under agitation using a magnetic field. Original video recorded using confocal microscopy. Total real-time duration is 10 s. Scale bar = 30 μm

Movie 2. Recording of capsules containing responsive particles, $\phi_{p,disp}$ = 3.0×10^{-2}. Particles are being agitated with an external magnetic field using a magnetic stirrer plate. 24 fps, Scale bar = 0.5 mm

Movie 3. Recording of macroscopic intensity change in capsules being agitated with a magnetic stirrer plate. 24 fps, Scale bar = 10 mm.

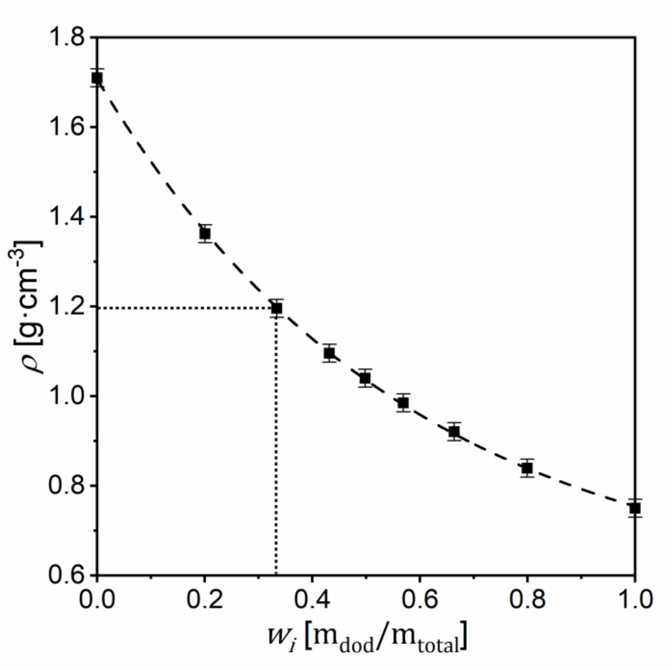

Figure S1. Binary solvent density curve calculated from volumetric experiments.

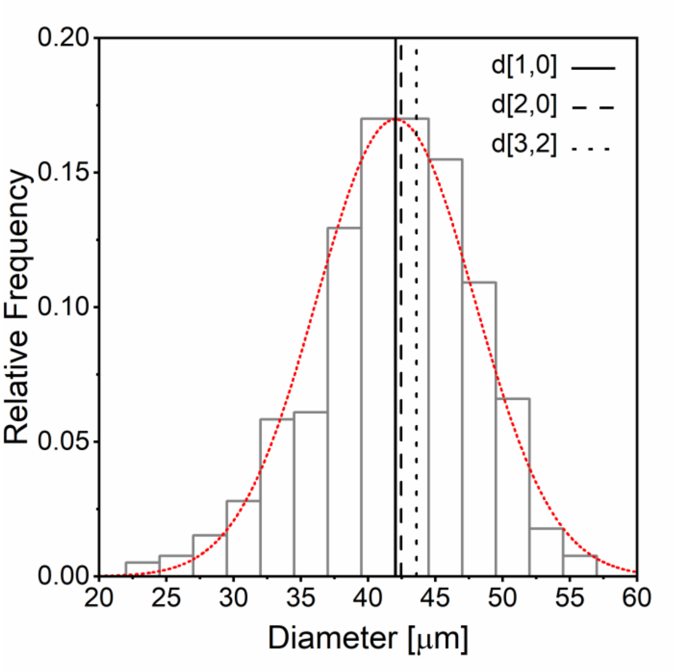

Figure S2. Size distribution of Janus particles as measured by different methods. Measured; d[3,2] 43.6 ± 5.9 μm. Graph and data resused from previous publication using same preparation and batch of particles (OA License CC BY 4.O, https://creativecommons.org/licenses/by/4.0/).[41]

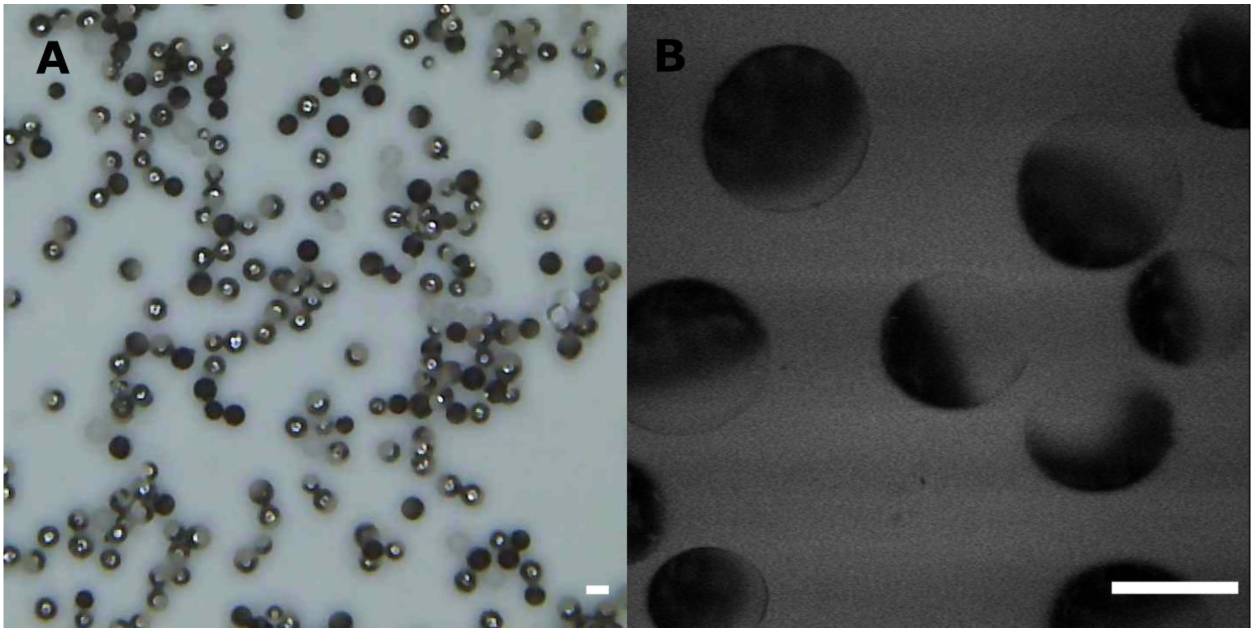

Figure S3. (a) Digital microscopy of PMMA Janus particles dried on a glass substrate. (b) Confocal microscopy image of the same PMMA Janus particles. Scale bars = 43.6 µm (1 average particle diameter from d[3,2]). Images and Data reused from previous publication using the same preparation and batch of particles (OA License CC BY 4.0, https://creativecommons.org/licenses/by/4.0/).[41]

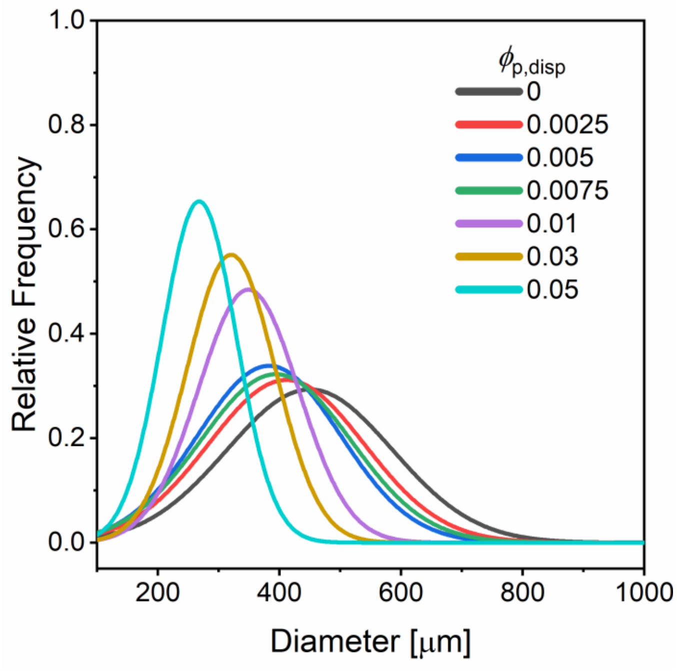

Figure S4. Size distribution of microcapsules formed using dispersed phases containing increasing volume fractions of Janus particles. Measured over at least 300 capsules for each data set, using a d[3,2] size distribution.

Table S1. Number of particles per capsule, N_p, volume fraction of particles per capsule, $\phi_{p,caps}$, and fraction of mobile particles per capsule, Ω_p, for all initial dispersed phase volume fractions from Table 1.

$\phi_{p,disp}$ ⸸	$\phi_{p,caps}$ [$\times 10^{-3}$] †	N_p ‡	Ω_p ⁑
0	-	-	-
2.5×10^{-3}	4.38 ± 3.0	3.62 ± 4.64	0.80 ± 0.36
5.0×10^{-3}	9.00 ± 6.0	5.66 ± 5.14	0.88 ± 0.29
7.5×10^{-3}	9.25 ± 7.0	6.13 ± 4.91	0.94 ± 0.23
1.0×10^{-2}	13.3 ± 6.0	6.64 ± 4.33	0.95 ± 0.19
3.0×10^{-2}	18.0 ± 9.0	7.31 ± 5.41	0.97 ± 0.15
5.0×10^{-2}	26.0 ± 15.0	6.58 ± 8.66	0.95 ± 0.19

⸸ Volume fraction of particles in the dispersed phase before emulsification. † Volume fraction of particles. ‡ Number of particles in a capsule. ⁑ Fraction of particles within a capsule which are mobile under external field agitation.

References

41. S. R. Wilson-Whitford, J. Gao, M. C. Roffin, W. E. Buckley and J. F. Gilchrist, Nat. Commun., 2023, 14, 5829.
